# Relationship between tooth loss and mortality in 80-year-old Japanese community-dwelling subjects

**DOI:** 10.1186/1471-2458-10-386

**Published:** 2010-07-01

**Authors:** Toshihiro Ansai, Yutaka Takata, Inho Soh, Shuji Awano, Akihiro Yoshida, Kazuo Sonoki, Tomoko Hamasaki, Takehiro Torisu, Akira Sogame, Naoko Shimada, Tadamichi Takehara

**Affiliations:** 1 Division of Community Oral Health Science, Department of Health Promotion, Kyushu Dental College, Kitakyushu, Japan; 2 Division of General Internal Medicine, Department of Health Promotion, Kyushu Dental College, Kitakyushu, Japan; 3 Division of Nutrition, Department of Home Economics, Kyushu Women's University, Kitakyushu, Japan; 4 Kurate Office for Health, Human Services and Environmental Issues, Nogata, Japan; 5 Kitakyushu Public Health and Welfare Bureau, Kyushu Dental College, Kitakyushu, Japan

## Abstract

**Background:**

Findings from several studies suggest associations between tooth loss and health outcomes, including malnutrition, poor quality of life, and mortality, in older individuals. However, limited information is available regarding whether those associations remain true in very elderly subjects after adequately considering confounding factors such as sex and smoking status. Herein, we determined whether the number of teeth in 80-year-old subjects is an independent predictor of mortality.

**Methods:**

We initially contacted 1282 80-year-old community-dwelling individuals born in 1917, of whom 697 responded and participated in a baseline study, with follow-up examinations conducted 4 and 5.5 years later. Data from interviews and medical and oral examinations were obtained, and oral health was determined according to the number of teeth remaining in the oral cavity.

**Results:**

A total of 108 and 157 subjects died in 4 years and 5.5 years, respectively, after the baseline study. Tooth loss was significantly associated with mortality at age 85.5, but not at age 84, after adjusting for potential confounders. When the analysis was stratified by sex, we found a stronger association in females in follow-up examinations conducted at both 4- and 5.5 years. On the other hand, the effect of tooth loss on mortality was not significantly different between smokers and non-smokers.

**Conclusion:**

Tooth loss is a significant predictor of mortality independent of health factors, socio-economic status, and lifestyle in octogenarians, with a stronger association in females.

## Background

Associations between tooth loss and mortality have been reported, though issues related to important confounding factors such as age, gender, and smoking status, which may be related to oral health and are also risk factors for mortality, remain to be clarified. In a number of studies, the age range of the subjects is broad [1-3], while there is a limited number of reports regarding the association of tooth loss and mortality in subjects who are the same age. An important merit of limiting a study population to the same age is that there is no need to consider the effects of age on changes that may potentially confound factors related to systemic condition. Thus, results obtained with such a study design would be expected to reveal a much higher level of scientific evidence. In a search of published studies regarding the association between tooth loss and mortality in a single age group, we found 3 reports: a 10-year cohort study of 80-year-old subjects by Hämalainen et al., in which the hazard ratio (HR) for number of missing teeth was 1.026 [4], an 18-year cohort study of 1803 70-year-old subjects by Österberg et al., in which the HRs for numbers of present teeth in males and females were 0.98 and 0.79, respectively [5], and an investigation by Holm-Pedersen et al. of 573 70-year-old Danish individuals examined every 5 years for 20 years, in which the HR for mortality 21 years after beginning the study in subjects who were edentulous at the age 70 was 1.26 [6].

Smoking is a major risk factor for oral diseases, including periodontitis and systemic diseases, as well as cardiovascular disease and aspiration pneumonia. However, there is concern regarding the effects of smoking status, as previously published reports included inadequate measurement and control for smoking status, and confounding factors and effect modification have become increasingly important. Furthermore, another important issue is the source of study subjects, as nearly all published studies investigated subjects in Western countries. To the best of our knowledge, only 1 report focused on a single-age non-Western population, in which a Japanese population-based cohort was investigated [7]. In that study, the subjects were elderly (mean age approximately 82 years old) and the age range was narrow, though the sample size was small (59 males, 59 females) and smoking status was based on information obtained at the age of 60.

In the present cohort study, we analyzed the association between number of teeth at the age of 80-years old and mortality after 4 and 5.5 years, and then evaluated the effects of sex and smoking status on that association.

## Methods

### Study population

A total of 1282 80-year-old candidates from 3 cities (Buzen, Yukuhashi, and Munakata), 4 towns (Katsuyama, Tsuiki, Toyotsu, and Kanda), 1 village (Shinyoshitomi), and 1 ward (Tobata of Kitakyushu City) in Fukuoka Prefecture on Kyushu Island in southern Japan were invited to participate. Those 9 locations were selected randomly from urban, suburban, and rural communities to achieve a balance of living environments in terms of socio-demographic backgrounds, dietary habits, health behaviors, and available medical care. This study was designed as an investigation of a representative population of individuals residing in the eastern area of Kyushu Island who were born in 1917. The percentage of 80-year-old individuals living in the study locations was approximately 0.62% of all residents, which was similar to the percentage (0.64%) of 80-year-old individuals residing in all of Kyushu. Of those 1282 individuals, 697 (54.4%) (277 males, 420 females) agreed to participate in the present study and completed a questionnaire regarding lifestyle, and oral and systemic health, and also underwent physical, laboratory blood, and oral examinations. The Human Investigations Committee of Kyushu Dental College approved the survey and all subjects gave written informed consent prior to participation.

### Baseline data

The baseline survey was performed in March 1998, during which time the participants took part in a face-to-face interview, and answered a questionnaire containing 37 questions about oral and systemic health status, use of medical (or dental) services, personal hygiene, healthcare practices (including smoking habit), and medical conditions [8]. Dental examinations were also performed using WHO criteria [9] by 3 trained dentists assisted by a dental nurse. In this study, only the number of teeth and denture status (fixed prostheses, partial or full dentures) were used for analysis. Remaining roots were not counted as retained teeth.

### Follow-up survey

This study utilized data obtained from 1998 to 2003 and was designed to analyze the results of follow-up examinations of all subjects in our previous study at the ages of 84 and 85.5 years old. Information on the survival of the subjects was collected from the registers at the Public Health Centers of each district included in the study. Of all subjects who took part in the baseline survey, there were no losses during the follow-up period, as even subjects who had moved away from Fukuoka Prefecture after the initial survey were successfully traced.

### Statistical analyses

Power analysis was performed using the software package G-power. The statistical power of this study was found to be 87%, with sample sizes of 540 for n1 and 157 for n2, an effect size of 0.25, and an α value of 0.05 set (two tailed t-test with accuracy mode), which demonstrated reasonable power. The main characteristics of participants who died versus those who remained alive during the follow-up period are reported as mean values (standard deviation, SD) or percentages. Differences between groups were tested using ANOVA for continuous variables and a Pearson chi-square test for categorical variables. Covariates determined at the baseline were sex, marital status, place of residence, medical history, laboratory blood test results (serum total cholesterol, fasting serum glucose, serum albumin), systolic and diastolic blood pressures, body mass index, regular physical activity, regular checkup by family doctor, self-rated general health, smoking habit, and daily alcohol consumption. Place of residence was included as a proxy for socioeconomic status.

The association between number of teeth and mortality was analyzed using multivariate logistic analysis. Preliminary analyses included univariate analyses of the association between number of teeth and mortality, covariates and number of teeth, and covariates and mortality. Multivariate analyses included variables that were related to number of teeth and mortality (P < 0.05) in bivariate analyses, which included gender, smoking status, serum total cholesterol, fasting serum glucose, serum albumin, place of residence, marital status, and body mass index. We sequentially tested the effect modification of tooth loss-mortality associations by adding interaction terms between tooth loss and sex, and smoking status according to likelihood ratio tests.

All statistical analyses were performed using SPSS 14.0 for Windows (SPSS, Chicago, Illinois, USA).

## Results

After 4 years, 108 of the subjects (58 males, 50 females) had died, while 157 (81 males, 76 females) had died by 5.5 years. Socio-demographic and medical characteristics of the study participants at the age of 80 are shown in Table 1. The deceased individuals had lower total cholesterol, higher glucose concentration, and lower serum albumin values as compared to those who remained alive, while BMI was higher in the survivors. Men and smokers were more likely to die during the follow-up period. The mortality rate was higher in males than in females at both 4 and 5.5 years, namely, 20.9% vs. 11.9%, and 29.2% vs. 18.1%, respectively. The dental characteristics of the subjects are shown in Table 2. Among the 697 subjects, the prevalence of edentulism was 33.9% (n = 236), and the proportion with 20 teeth or more was 15.2% (n = 106). As compared to edentulous males, edentulous females had a 1.3-fold higher rate of mortality in the 4-year follow-up examinations and a 1.2-fold higher rate of mortality in the 5.5-year follow-up examinations. The number of teeth (or number of missing teeth) and health-related behaviors such as brushing habit and regular check-ups by dentists differed between the survivors and deceased subjects.

**Table 1:** Sociodemographic and medical characteristics of 80-year-old subjects based on survival during 5.5-year follow-up period

Characteristic	Alive	Died	P value
	(n = 540)	(n = 157)	
Sex			
Female	344 (81.9%)	76 (18.1%)	0.001
Male	196 (70.8%)	81 (29.2%)	
Marital status^a^			
Currently married	253 (74.2%)	88 (25.8%)	0.022
Currently unmarried	268 (80.9%)	63 (19.1%)	
Place of residence^b^			
Ward	149 (85.6%)	25 (14.4%)	0.008
City	215 (76.2%)	67 (23.8%)	
Town/village	170 (73.3%)	62 (26.7%)	
Medical history			
CVD	90 (76.3%)	28 (23.7%)	0.358
Cancer	2 (66.7%)	1 (33.3%)	0.528
Pneumonia	13 (72.2%)	5 (27.7%)	0.364
Medical examinations			
Serum total cholesterol (mg/dL)	208.1 (37.4)	196.8 (40.1)	0.002
Fasting serum glucose (mg/dL)	116.5 (44.5)	138.9 (74.6)	< 0.001
Serum albumin (g/dL)	4.3 (0.29)	4.1 (0.35)	< 0.001
Systolic blood pressure (mmHg)	150.8 (23.6)	149.2 (21.8)	0.463
Diastolic blood pressure (mmHg)	79.3 (12.5)	77.5 (11.7)	0.117
Disease risk factors			
Body mass index	22.9 (3.3)	21.9 (3.2)	0.002
Regular physical activity^c^			
Yes	300 (77.9%)	85 (22.1%)	0.614
No	212 (76.3%)	66 (23.7%)	
Regular checkup by family doctor^d^			
Yes	430 (77.5%)	125 (22.5%)	0.688
No	81 (75.7%)	26 (24.3%)	
Self-rated general health^e^			
Very good	224 (79.2%)	59 (20.8%)	0.140
Good	245 (78.3%)	68 (21.7%)	
Moderate	61 (69.3%)	27 (30.7%)	
Smoking habit^c^			
Smoker	173 (68.4%)	80 (31.6%)	< 0.001
Non-smoker	358 (82.9%)	74 (17.1%)	
Daily alcohol consumption^f^			
Non-drinker	221 (75.4%)	72 (24.6%)	
Drinker	294 (79.0%)	78 (21.0%)	0.156

Abbreviations: CVD, cardiovascular disease.

Data indicate the number of subjects (%) or mean (SD).

Data available for ^a^672, ^b^688, ^c^663, ^d^662, ^e^684, and ^f^665 people, respectively.

Differences between groups were tested using a Pearson chi-square test for categorical variables and an ANOVA for continuous variables.

**Table 2:** Dental characteristics of 80-year-old subjects based on survival during 5.5-year follow-up period

Characteristic	Alive	Died	P value
	(n = 540)	(n = 157)	
Number of teeth^a^	8.4 (9.0)	6.7 (8.2)	0.031
Number of missing teeth^a^	23.5 (9.0)	25.3 (8.2)	0.026
Number of edentulous subjects^a^	178 (75.4%)	58 (24.6%)	0.196
Number of dentate subjects	353 (78.6%)	96 (21.4%)	
Number of teeth & denture status^b^			
No denture	89 (83.9%)	17 (16.1%)	0.108
PDs	114 (80.9%)	27 (19.1%)	
Either PD or FD	142 (74.3%)	49 (25.7%)	
FDs	164 (73.9%)	58 (26.1%)	
Self-care^c^			
Yes	400 (78.7%)	108 (21.3%)	0.091
No	103 (72.1%)	40 (27.9%)	
Toothbrushing^d^			
≥ 2 times/day	240 (83.6%)	47 (16.4%)	0.004
< 2 times/day	213 (73.7%)	76 (26.3%)	
Regular checkups by dentist^e^			
Yes	360 (79.6%)	92 (20.4%)	0.027
No	151 (71.9%)	59 (28.1%)	

Abbreviations: FD, full denture; PD, partial denture (including fixed prostheses).

Data indicate the number of subjects (%) or mean (SD).

Data available for ^a^685, ^b^660, ^c^651, ^d^576, and ^e^662 people, respectively.

Differences between groups were tested using a Pearson chi-square test for categorical variables and an ANOVA for continuous variables.

Table 3 shows adjusted odds ratios (ORs) for number of teeth in regard to mortality at the 4-year follow-up examination. The overall OR was 0.980 [95% confidence interval (CI), 0.953-1.007]. We performed interaction tests to investigate whether sex and smoking status were associated with tooth loss, and found a significant association between the risk of tooth loss and sex (P = 0.006), while there was no such association between smoking status and tooth loss. Accordingly, it was suggested that sex should be treated as an effect modifier in the following analyses. When stratified by sex, only the OR for number of teeth in females was significant (0.937, 95% CI, 0.889-0.987), while there was not a significant association in males (OR, 1.004, 95% CI, 0.969-1.040).

**Table 3:** Odds ratios for effect of number of teeth on mortality at 4-year follow-up examination according to sex: Multivariate logistic regression analysis

			OR by sex^c^			
			
	Adjusted OR (95% CI)^a^	P		Cases	Adjusted OR (95% CI)^b^	P
Number of teeth	0.980 (0.953-1.007)	0.148	Males	277	1.004 (0.969-1.040)	0.841
			Females	420	0.937 (0.889-0.987)	0.014

OR: odds ratio, CI: confidence interval.

^a^Adjusted for sex, smoking habit, serum total cholesterol, fasting serum glucose, serum albumin, place of residence, marital status, and body mass index.

^b^Adjusted for smoking habit, serum total cholesterol, fasting serum glucose, serum albumin, place of residence, marital status, and body mass index.

^c^Stratified by sex due to results of interaction tests, which indicated a statistically significant difference for risk related to tooth loss between males and females (P = 0.006) (see statistical analyses).

Table 4 shows adjusted ORs for number of teeth in regard to mortality at the 5.5-year follow-up examination. The overall OR for number of teeth was 0.972 (95% CI, 0.948-0.996). Furthermore, the adjusted OR for female gender was 0.946 (95% CI, 0.907-0.987), while that for male gender was 0.986 (95% CI, 0.955-1.019), which was not significantly different.

**Table 4:** Odds ratios for effect of number of teeth on mortality at 5.5-year follow-up examination according to sex: Multivariate logistic regression analysis

			OR by sex^c^			
			
	Adjusted OR (95% CI)^a^	P		Cases	Adjusted OR (95% CI)^b^	P
Number of teeth	0.972 (0.948-0.996)	0.023	Males	277	0.986 (0.955-1.019)	0.399
			Females	420	0.946 (0.907-0.987)	0.011

OR: odds ratio, CI: confidence interval.

^a^Adjusted for sex, smoking habit, serum total cholesterol, fasting serum glucose, serum albumin, place of residence, marital status, and body mass index.

^b^Adjusted for smoking habit, serum total cholesterol, fasting serum glucose, serum albumin, place of residence, marital status, and body mass index.

^c^Stratified by sex due to results of interaction tests, which indicated a statistically significant difference for risk related to tooth loss between males and females (P = 0.006) (see statistical analyses).

## Discussion

In the present longitudinal study, we investigated associations between tooth loss and mortality in 80-year-old Japanese subjects, and obtained the following important findings: (1) tooth loss was associated with higher mortality in our 80-year-old subjects; (2) the associations differed due to sex, with female gender significantly associated with mortality; and (3) smoking status did not affect the association between tooth loss and mortality. Abnet et al. [1] reported a difference between genders for the association between tooth loss and mortality, as that association was stronger in males, though the difference between females and males was only slight. Also, 2 different reports presented by Japanese researchers found an association between the number of teeth and mortality in elderly males [2,7]. Their findings did not coincide with ours, though the reasons for the differences remain unclear. They might be partly explained by the different age range and life-style related factors of the study subjects.

There are a number of reports regarding the association between number of teeth and mortality. However, some of those analyzed subjects with a broad age range, used a small number of subjects, studied mixed genders, and/or investigated subjects with smoking habit information lacking. In a literature search, we found only 3 reports of a cohort study of the association between number of teeth and mortality in a single age elderly population [4-6]. The baseline ages of the subjects in those reports were either 80 or 70 years old, and they presented similar conclusions stating that tooth loss is independently associated with mortality and independent of other potential confounding factors. On the other hand, treatment of smoking habit varied among those 3 reports, as Hämalainen et al. [4] did not provide adequate information regarding smoking status, while the others indicated smoking status, but did not show the effects of smoking [5,6]. An explanation for our result regarding the role of smoking status may be related to survivor effects. In our subjects who survived to the age of 85.5, the effect of smoking may have been reduced.

The mechanism of the association between tooth loss and mortality has been hypothesized to consist of 2 pathways; an infection and inflammation pathway, and a nutritional pathway, as reported by Janket et al. [10]. Tooth loss is known to mainly be caused by periodontal disease, the most common oral infectious disease, indicating an inflammatory burden from a past infection, and periodontitis is associated with a steady increase in circulatory levels of pro-inflammatory cytokines [11,12]. In addition, inadequate dentition due to tooth loss may affect eating behavior, including mastication and food choice, causing individuals to substantially reduce their intake of fruits, vegetables, and other key nutrients [13], which have been shown to be associated with increased survival and lower cardiovascular mortality [14]. Furthermore, adults who have no natural teeth and wear complete replacement dentures tend to ingest a high fat, low fiber diet [15]. However, we have no direct evidence to support any of those speculations at the present time.

A limitation of our study is that the sample consisted largely of generally healthy elderly subjects, who might have been more eager and/or able to participate. Thus, our findings may indicate the association in generally healthy elderly subjects. The response rate to participate in the study was 54%, which was related to location and health-oriented behavior, as individuals who resided in towns, and those who regularly attended check-up examinations by a family doctor or dentist had higher response rates. On the other hand, several findings have been presented regarding the effect of inadequate dental status on mortality of institutionalized elderly individuals [16], in which poor dentition was found to be associated with high overall mortality, thus systematic attention to dental status is recommended.

## Conclusions

In this study, our results showed a significant relationship between tooth loss and mortality in 80-year-old individuals, even after extensive adjustment for potential confounding variables. Since a stronger association was found in females, we recommend that researchers include gender when performing related statistical analyses. Our findings also have significant implications in terms of public health strategy, because they indicate that improving oral health and keeping more natural teeth will increase the health status of the population as well as longevity.

## Competing interests

The authors declare that they have no competing interests.

## Authors' contributions

The epidemiological study was supervised by TT and TA. TA, YT, IS, SA, AY, KS, TH, TT, AS, NS, and TT participated in the epidemiological study. TT initially designed the study. TA contributed toward analysis and interpretation of data, and wrote the first draft of the manuscript. All authors read and approved the final version of the report.

## Pre-publication history

The pre-publication history for this paper can be accessed here:

http://www.biomedcentral.com/1471-2458/10/386/prepub
